# Novel pathogenic alterations in pediatric and adult desmoid-type fibromatosis – A systematic analysis of 204 cases

**DOI:** 10.1038/s41598-020-60237-6

**Published:** 2020-02-25

**Authors:** Marcel Trautmann, Jan Rehkämper, Heidrun Gevensleben, Jessica Becker, Eva Wardelmann, Wolfgang Hartmann, Inga Grünewald, Sebastian Huss

**Affiliations:** 1 Gerhard-Domagk-Institute of Pathology, Münster University Hospital, Münster, Germany; 2 Division of Translational Pathology, Gerhard-Domagk-Institute of Pathology, Münster University Hospital, Münster, Germany; 3 Institute of Pathology, Bonn University Hospital, Bonn, Germany; 4 Institute of Human Genetics, University of Bonn, School of Medicine & Bonn University Hospital, Bonn, Germany

**Keywords:** Next-generation sequencing, Cancer genomics, Targeted therapies, Sarcoma, Genetic testing

## Abstract

Desmoid-type fibromatosis (DTF, aggressive fibromatosis) is a non-metastasizing mesenchymal neoplasm of deep soft tissue with a tendency towards local recurrence. Genetic alterations affecting canonical Wnt/β-catenin signaling are reported in the majority of DTF. While most sporadic DTF harbor somatic mutations in *CTNNB1*, germline mutations in adenomatous polyposis coli (*APC)* are known to occur in hereditary DTF types (FAP, Gardner-Syndrome). Additional single nucleotide variants (SNVs) in *AKT1* (E17K) and *BRAF* (V600E) were reported in pediatric DTF with potential clinical implications. We performed targeted next-generation sequencing (NGS) in a large cohort of 204 formalin-fixed DTF samples, comprising 22 pediatric cases (patients age ≤18 years). The mutational status was correlated with clinicopathological characteristics. Overall, deleterious *CTNNB1* mutations were detected in 89% of DTF, most frequently affecting the serine/threonine phosphorylation sites T41 and S45 of β-catenin. While the T41A *CTNNB1* mutation was significantly more often identified in the mesenterial localization, DTF originating from extra-intestinal sites more frequently harbored the S45P *CTNNB1* alteration. Beyond common mutations in *CTNNB1*, additional SNVs were demonstrated in 7% of the DTF cohort and in 18% of the pediatric DTF subgroup. The mutational spectrum included deleterious mutations in *AKT1* (G311S/D and T312I), *ALK* (R806H and G924S), *AR* (A159T), *EGFR* (P848L), *ERBB2* (H174Y), *IDH2* (H354Y), *KIT* (V559D), *RET* (T1038A), *SDHA* (R325M), and *SDHD* (R115W), as characterized by *in silico* prediction tools. In conclusion, our study indicates that DTF may harbor a broader mutational spectrum beyond *CTNNB1* mutations, comprising targetable alterations including the herewith first reported imatinib-sensitive *KIT* V559D mutation in DTF.

## Introduction

Desmoid-type fibromatosis (aggressive fibromatosis, desmoid tumor, DTF) is an infiltrating, locally aggressive myofibroblastic neoplasm of intermediate malignant potential highly prone to local recurrence without the potential for metastatic spread. The tumors typically arise in deep soft tissue compartments of intra- and extra-abdominal localization. Pediatric forms usually affect the extremities, while in adults also the mesenterium and the abdominal wall are commonly involved sites; the latter predominantly affected in women^1^.

The majority of DTF harbor mutations affecting the canonical Wnt/β-catenin signaling pathway^2^. While in patients with familial adenomatous polyposis (FAP) β-catenin is not degraded through inactivating mutations in *APC*, most sporadic DTF harbor alterations in *CTNNB1*; both leading to a nuclear accumulation of β-catenin and an oncogenic activation of the Wnt/β-catenin signal transduction pathway^3–6^. In the sporadic subtype, alterations in *CTNNB1* seem to be focused on the serine/threonine phosphorylation sites T41 and S45^7,8^ with a higher risk of local recurrence reported in association with the S45F *CTNNB1* mutation^8,9^.

Currently, no evidence-based approach for the treatment of DTF is established^10^ and the clinical course is still unpredictable with spontaneous regression as well as progression and long-lasting stable disease being reported for individual cases. Given the broad spectrum of clinical presentation, initial treatment decisions are highly individualized. However, primary en bloc surgery is no longer regarded as mandatory, as high recurrence rates (up to 60%) have been reported^11^. Currently, a shift towards a “wait and see” strategy is assessed by selected clinical trials and recommended by different studies^10,12,13^. In a retrospective study, Penel *et al*. reported no significant difference with regard to the outcome between surgical *vs*. non-surgical treatment approaches^14^. However, these results still have to be evaluated in prospective trials.

Several trials evaluated the benefit of additional systemic therapies in DTF. The multitargeted receptor tyrosine kinase inhibitor sorafenib prolonged progression-free survival in a double-blind phase 3 trial including 87 patients with progressive, symptomatic or recurrent DTF^15^. Furthermore, in a phase 2 study, the tyrosine kinase inhibitor imatinib was shown to represent a treatment option in *CTNNB1* mutated DTF, especially in tumors carrying the S45F alteration^16^. Another phase 2 study reported on the efficacy of the Notch pathway inhibitor PF-03084014 in patients with pre-treated, progressive and symptomatic DTF^17^. The rationale to inhibit the Notch signaling pathway is based on the demonstration of signaling cross talk between the Notch- and the Wnt/β-catenin pathways^17,18^. There seem to be several intrinsic (i.e. mutational status or DTF tumor localization) and extrinsic (i.e. initial treatment decision) factors that may influence the biology of the disease.

The majority of genetic studies published so far applied conventional Sanger sequencing of *CTNNB1* (limited to exon 3), focusing predominantly on the mutational subtype of sporadic DTF. Recently, Meazza *et al*. analyzed a small cohort of DTF employing NGS for mutational analysis and reported potential clinical implications for pediatric (patients age ≤18 years) DTF harboring gain-of-function mutations in *AKT1* and *BRAF*^19^. These findings suggest a more complex mutational spectrum of DTF than expected so far, which may potentially be associated with therapeutic implications.

In our present genetic study, we performed an NGS-based comprehensive molecular characterization of 204 DTF cases comprising approximately 10% of pediatric samples. Genotyping results were correlated with clinical and pathological features with special emphasis on the mutational spectrum beyond *CTNNB1* in pediatric and adult DTF.

## Results

### Spectrum of *CTNNB1* alterations in DTF samples

**Figure 1:**
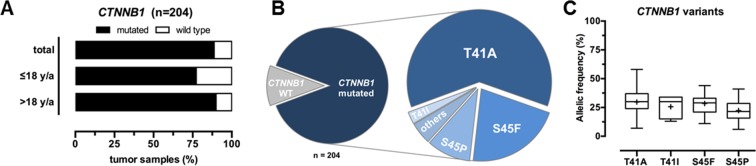
(**A**) Frequency of detected *CTNNB1* mutations in the pediatric and adult DTF subgroup (n = 204). (**B**) Spectrum of detected *CTNNB1* mutations. (**C**) Range of allelic frequencies for the most common *CTNNB1* mutation subtypes.

As pathogenic mutations in the *CTNNB1* gene can be responsible for the constitutive activation of the canonical Wnt/β-catenin signaling cascade, we analyzed the entire *CTNNB1* coding region by targeted NGS followed by validation via Sanger sequencing. Overall, the complete cohort of 204 DTF cases was successfully analyzed (Table 1 and Supplementary Table S1). In total, deleterious *CTNNB1* mutations were detected in n = 181/204 (88.7%) DTF samples, with a minor fraction detected in the pediatric (77.3%) compared to the adult (89.9%) subgroup (Fig. 1A and Table 2). All four tumors in patients with familial adenomatous polyposis (FAP) analyzed in this study, were assigned to the *CTNNB1* wild type group (n = 23/204; 11.3%). The majority of deleterious alterations in the *CTNNB1* gene were restricted to the serine/threonine phosphorylation sites T41 and S45, including T41A (n = 111/204; 54.4%), S45F (n = 40/204; 19.6%), S45P (n = 18/204; 8.8%) or T41I (n = 5/204; 2.5%) amino acid exchanges (Fig. 1B and Table 2). Detected allelic frequencies were in the range from minimal 6% (S45P) to 13% (T41I) and maximal 34% (T41I) to 58% (T41A) (Fig. 1C). Less frequent *CTNNB1* mutations were detected in individual DTF cases in the adult subgroup, comprising S33T (n = 1/204), S33L (n = 1/204), G34R (n = 1/204) and S45KR (n = 1/204) amino acid exchanges. In addition, biallelic mutations (S45T/S45Y, S45T/S45F and S45P/S45F) were identified in four adult samples. Comparing the pediatric (≤18 y/a) to the adult (>18 y/a) age groups, the fraction of *CTNNB1* wildtype cases was larger in the pediatric (n = 5/22; 22.7%) *vs*. the adult (n = 18/170; 10.5%) group. Deleterious T41A (n = 97/170; 56.7% *vs*. n = 8/22; 36.4%) and S45P (n = 16/170; 9.4% *vs*. n = 1/22; 4.5%) β-catenin alterations were detected more often in adult DTF patients, whereas three out of four T41I variants were demonstrated in the pediatric subgroup (n = 1/170; 0.6% *vs*. n = 3/22; 13.6%). All pediatric DTF cases were localized in extra-intestinal sites in contrast to the frequent occurrence of extra-intestinal localization in adult DTF cases (n = 20/20; 100% *vs*. n = 86/164; 52.4%, p < 0.05).

**Table 1:** Clinicopathological patient and tumor characteristics.

	No. of patients (%)		
	Total n = 204	Pediatric (≤18 y/a) n = 22 (11)	Adult (>18 y/a) n = 170 (89)
**Age (years)**			
Mean [±SD]	41.1 [±19]	11.9 [±6]	44.9 [±17]
Median [range]	40.5 [0–82]	15.0 [0–18]	43.0 [19–82]
**Type**			
Sporadic	200 (98)	21 (95)	167 (98)
Syndromal	4 (2)	1 (5)	3 (2)
**Gender**			
Female	118 (61)	14 (64)	104 (61)
Male	74 (39)	8 (36)	66 (39)
**Tumor size (cm)**			
Mean [±SD]	8.0 [±5]	6.2 [±3]	8.2 [±5]
Median [range]	7.0 [1–28]	5.3 [3–13]	7.5 [1–28]
**Tumor site**			
I. Extra-intestinal	106 (58)	20 (100)	86 (52)
a. Head and neck	13	5	8
b. Trunk	47	3	44
c. Upper extremities	11	3	8
d. Lower extremities	32	9	23
II. Intra-abdominal	59 (32)	0 (0)	59 (36)
III. Abdominal	19 (10)	0 (0)	19 (12)

In total, clinicopathological data for analysis were available for ≥90% of patients with DTF. The entire cohort of 204 patients was used for the NGS-based genotype study. Abbreviations: y/a, years of age; SD, standard deviation; ND, not determined.

**Table 2:** Clinicopathological patient and tumor characteristics in accordance with *CTNNB1* genotype.

	No. of patients (%)				
	*CTNNB1* wild type	∆S33	∆G34	∆T41	∆S45
**Age (years)**					
≤18 y/a	5 (22)	—	—	11 (10)	6 (10)
>18 y/a	18 (78)	2 (100)	1 (100)	98 (90)	52 (90)
**Type**					
Sporadic	19 (83)	2 (100)	1 (100)	116 (100)	63 (100)
Syndromal	4 (17)	—	—	—	—
**Gender**					
Female	15 (65)	1 (50)	1 (100)	68 (62)	34 (59)
Male	8 (35)	1 (50)	—	41 (38)	24 (41)
**Tumor size (cm)**					
≤8.0	7 (78)	2 (100)	1 (100)	35 (57)	14 (61)
>8.0	2 (22)	—	—	26 (43)	9 (39)
**Tumor site**					
I. Extra-intestinal	15 (68)	—	—	53 (50)	39 (71)
a. Head and neck	3	—	—	7	3
b. Trunk	7	—	—	24	17
c. Upper extremities	0	—	—	8	3
d. Lower extremities	4	—	—	13	15
II. Intra-abdominal	5 (23)	1 (50)	—	43 (41)	10 (18)
III. Abdominal	2 (9)	1 (50)	1 (100)	10 (9)	6 (11)

In total, clinicopathological data for analysis were available for ≥90% of patients with DTF. The entire cohort of 204 patients was used for the NGS-based genotype study. Abbreviations: wt, wild type (not mutated); y/a, years of age.

Correlating the individual *CTNNB1* mutational status and tumor site, we observed a significantly higher incidence of deleterious T41 alterations in intra-abdominal compared to abdominal and extra-abdominal DTF cases (n = 43/59; 72.9% *vs*. n = 63/125; 50.4%; p = 0.0042). In addition, β-catenin mutations affecting S45 were detected significantly more frequently in extra-abdominal cases than in intra-abdominal and abdominal DTF cases (n = 36/106; 34.0% *vs*. n = 16/78; 20.51%, p = 0.0222) (Table 2). Concerning a possible prognostic relevance of the mutational subtype, recurrences were reported in 18 cases, occurring in extra-intestinal localization in 17 cases.

**Figure 2:**
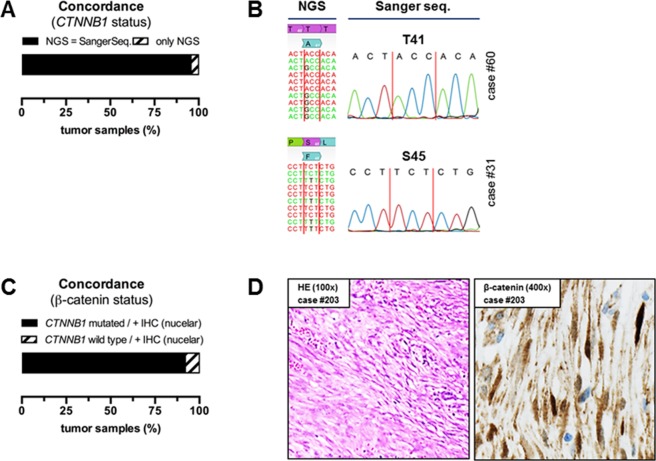
(**A**) Concordance of *CTNNB1* mutational status comparing NGS and Sanger sequencing results. (**B**) Representative visualization of NGS and Sanger sequencing results (case #60 and #31). (**C**) Concordance of *CTNNB1* mutational status and nuclear β-catenin immunoreactivity. (**D**) Representative HE and nuclear β-catenin IHC staining (case #203).

Only n = 6/131 (4.3%) *CTNNB1* mutations detected by NGS could not be validated by Sanger sequencing due to low allelic frequencies in the range of 6.0–17.6% (Fig. 2A,B). Tumor tissue for immunohistochemical analysis was available in 131 DTF cases. Nuclear β-catenin positivity correlated with the *CTNNB1* mutational status in 92.4% (n = 121/131) of DTF cases (Fig. 2C,D).

In three cases, more than one missense mutation was detected in *CTNNB1* (Supplemental Table S1). A 26-year old female with a DTF localized at the trunk (extra-intestinal) harbored both a S45T and a S45Y mutation in *CTNNB1* (case #26). The combination of S45F and S45T *CTNNB1* mutations was detected in a 32-year old female patient with abdominal DTF of unknown size (case #149). A combination of uncommon S33T and G34R *CTNNB1* alterations was detected in a 43-year old female patient with a 4.8 cm abdominal DTF (case #119). No additional non-*CTNNB1* mutations were detected by NGS in these cases.

To the best of our knowledge, two uncommon alterations were detected in *CTNNB1*: (I) S33L (97_98delinsCT) in an intra-abdominal DTF of a 60-year old male without additional mutations (case #181) and (II) S45KA in a 74-year old male with an abdominal 10.5 cm DTF (case #51). Both variants were predicted as potentially deleterious by *in silico* analysis.

### Spectrum of non-*CTNNB1* alterations in DTF samples

**Figure 3:**
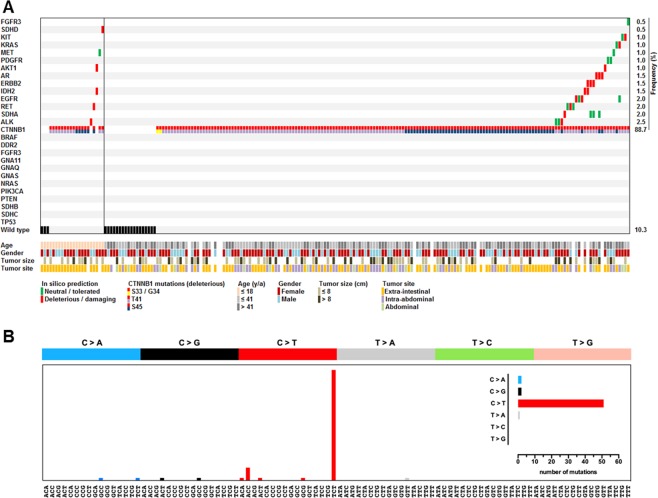
(**A**) Clustered mutational profile of pediatric and adult DTF (n = 204). Alterations in different genes (rows) are indicated for each sample (columns). Clinicopathological information and *in silico* prediction results on the potential deleterious impact of detected gene variants are summarized according to the legend. (**B**) Spectrum of single nucleotide substitutions identified in DTF and defined by the sequence context immediately 3′ and 5′ to the mutated nucleobase.

All protein coding exons of 26 additional cancer-associated genes were analyzed by NGS including: *AKT1, ALK, AR, BRAF, DDR2, EGFR, ERBB2, FGFR3, GNA11, GNAQ, GNAS, IDH1, IDH2, KIT, KRAS, MET, NRAS, PDGFRA, PIK3CA, PTEN, RET, SDHA, SDHB, SDHC, SDHD* and *TP53*. In 31 out of 204 (15.2%) DTF cases, additional non-*CTNNB1* alterations were detected (summarized in Fig. 3A,B and Supplementary Tables S1 and S2). No genetic alterations were identified in n = 3/22 (13.6%) pediatric and n = 18/170 (10.6%) adult DTF cases. Overall, 56.8% (n = 21/37) of the non-synonymous variants (in *AKT1, ALK, AR, EGFR, ERBB2, IDH2, KIT, KRAS, RET, SDHA* and *SDHD*) were predicted to have a potentially deleterious impact by ≥3 independent *in silico* tools (Supplementary Table S3). In contrast, all five variants identified in *FGFR3, MET and PDGFR* were classified *in silico* as potentially neutral/tolerated. Notably, deleterious mutations were more often demonstrated simultaneously to *CTNNB1* alterations affecting the phosphorylation site T41 compared to S45 (n = 13/21; 61.9% *vs*. n = 5/21; 23.8%; p = 0.0278) with three deleterious mutations exclusively identified in two *CTNNB1* wild type pediatric DTF cases. In total, deleterious non-*CTNNB1* mutations were demonstrated in n = 4/22 (18.2%; in *AKT1, ALK, IDH2, RET* and *SDHD*) pediatric *vs*. n = 15/170 (8.8%; in *AKT1, ALK, AR, EGFR, ERBB2, IDH2, KIT, KRAS, RET* and *SDHA*) adult DTF cases.

In one case (case #202), an imatinib-sensitive *KIT* (V559D) mutation was detected in addition to the *CTNNB1* (T41A) mutation. The tumor was localized in the Musculus rectus abdominis of a 43-year old female patient and displayed a diffuse infiltrative growth.

A combined S45P and S45F mutation was detected in a 25-year old male with an 8.0 cm DTF affecting the trunk (case #37). In this tumor, additional mutations were identified in *AR* (A159T) and *SDHA* (T36I). *In silico* analysis of the additional mutations indicated no pathogenic potential. Of note, in one case, a similar combination of S45P and S45F *CTNNB1* alterations and an A159T mutation in *AR* was detected with no clinical data available (case #38).

The known *KIT* polymorphism M541L was demonstrated in 16% of the analyzed DTF cases. Analyzing the mutational spectrum of single nucleotide substitutions from all n = 183/204 mutated DTF samples, C > T substitution was identified as major process (Fig. 3B).

## DISCUSSION

In this study, we attempted to refine the mutational spectrum in DTF beyond the well-established alterations affecting the canonical Wnt/β-catenin signaling pathway^2–6^. We conducted a comprehensive molecular characterization of 204 DTF cases applying NGS.

Based on our previous results analyzing mesenteric DTF^20^, we first examined the mutational *CTNNB1* status in correlation with clinicopathological features. Previous studies reported genomic *CTNNB1* alterations in 67–92% of sporadic DTF cases demonstrated by conventional Sanger sequencing and limited to *CTNNB1* exon 3^7–9,19–21^ (summarized in Table 3). In our present study, we conducted NGS with an increased sensitivity (≥10% allelic frequency) and detected deleterious *CTNNB1* mutations in an enlarged fraction of n = 181/204 (89%) DTF cases. Due to low allelic frequencies, 4.3% of *CTNNB1* mutations were exclusively traceable by NGS (The mean region coverage depth was 377×) and not detected by Sanger sequencing. With regard to clinical data, 4 cases (one pediatric) were known to be associated with FAP and harbored, as expected, no mutation in *CTNNB1*.

**Table 3:** *CTNNB1* mutational spectrum in selected previous studies of patients with DTF (cohort n > 20).

Study	Age	n (%)	*CTNNB1* mutational status, n (%)					
			wild type	∆*CTNNB1*	T41A	S45F	S45P	Others
Current study, 2020	Pediatric (≤18 y/a)	22 (11)	5	17	8	5	1	3
	Adult (>18 y/a)	170 (89)	18	152	97	32	16	7
	**Total**	**204**	**23 (11)**	**181 (89)**	**111 (54)**	**40 (20)**	**18 (9)**	**12 (6)**
Meazza *et al*.^18^	Pediatric (≤18 y/a)	28	10	18	8	6	5	—
	Adult (>18 y/a)	33	10	23	12	7	4	—
	Total	**61**	**20**	**41(67)**	**20**	**13**	**9**	—
Crago *et al*.^2^	Pediatric (≤18 y/a)	ND						
	Adult (>18 y/a)	ND						
	**Total**	**117**	**9 (8)**	**108 (92)**	**61 (52)**	**35 (30)**	**8 (7)**	**4 (3)**
Colombo *et al*.^9^	Pediatric (≤18 y/a)	ND						
	Adult (>18 y/a)	ND						
	**Total**	**179**	**48 (27)**	**131 (73)**	**80 (45)**	**39 (22)**	**12 (7)**	—
Huss *et al*.^19^	Pediatric (≤18 y/a)	ND						
	Adult (>18 y/a)	ND						
	**Total**	**84**	**13 (15)**	**71 (85)**	**58 (69)**	**5 (6)**	**5 (6)**	**3 (4)**
Dômont *et al*.^8^	Pediatric (≤18 y/a)	ND						
	Adult (>18 y/a)	ND						
	**Total**	**155**	**26 (17)**	**129 (83)**	**64 (41)**	**53 (34)**	**9 (6)**	**3 (2)**
Lazar *et al*.^7^	Pediatric (≤18 y/a)	ND						
	Adult (>18 y/a)	ND						
	**Total**	**138**	**21(15)**	**117 (85)**	**69 (50)**	**39 (28)**	**9 (7)**	—
Amary *et al*.^27^	Pediatric (≤18 y/a)	ND						
	Adult (>18 y/a)	ND						
	**Total**	**76**	**10(13)**	**66 (87)**	**27 (36)**	**34 (45)**	**5 (7)**	—

Abbreviations: y/a, years of age; ND, not determined.

**Figure 4:**
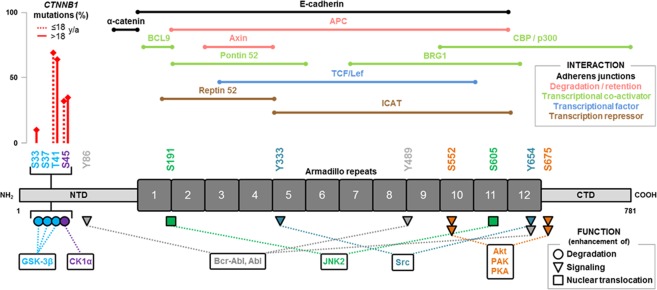
Schematic overview of β-catenin indicating relevant protein domains, phosphorylation sites, interaction partners and associated cellular functions.

As reported by previous studies, the majority of deleterious alterations in the *CTNNB1* gene were restricted to the serine/threonine phosphorylation sites T41 and S45. In our study, T41A (n = 111/204; 54.4%), S45F (n = 40/204; 19.6%) and S45P (n = 18/204; 8.8%) were the most frequent amino acid exchanges affecting sites involved in the modulation of the interaction of β-catenin with several kinases: Sequential phosphorylation at T41, S33 and S37 is mediated by the glycogen synthase kinase 3 beta (GSK-3β), while S45 is phosphorylated by the casein kinase-1 alpha (CK1α) targeting β-catenin for ubiquitination and subsequently proteasomal degradation^5,22,23^. Mutations affecting these phosphorylation sites lead to stabilization and translocation of β-catenin into the nucleus where it acts as a transcriptional regulator. A schematic overview of β-catenin indicating relevant protein domains, phosphorylation sites, interaction partners and associated cellular functions is shown in Fig. 4.

Several genotype-phenotype correlations have been published so far with the S45F alterations found to predict a higher tendency for local recurrence in DTF^7–9^. In accordance with results published by Colombo *et al*., we detected *CTNNB1* mutations in association with S45 significantly more often in extra-abdominal than in intra-abdominal or abdominal DTF cases (n = 36/106; 36.0% *vs*. n = 16/78; 20.51%, p = 0.0222). As previously indicated for mesenteric DTF^20^, we observed a significantly higher incidence of deleterious T41 alterations in intra-abdominal compared to abdominal and extra-abdominal DTF cases (n = 43/59; 72.9% *vs*. n = 63/125; 50.4%; p = 0.0042). Recently, the French Sarcoma group published data from a nationwide prospective study indicating that tumor localization constitutes a major prognostic factor. Two-year event free survival was significantly higher in favorable localizations than in unfavorable sites which was mainly extra-intestinal localization (except the lower limb)^14^.

We observed phenotypic and genetic differences comparing the pediatric and adult DTF groups. All pediatric DTF were localized extra-intestinal (100%; n = 20/20), while only 52.4% (n = 86/164) of DTF in adult patients occurred extra-intestinal. With regard to the data from the French Sarcoma group, an increased risk for local recurrence is implicated for the pediatric DTF group. For both DTF subgroups, the majority of deleterious alterations in the *CTNNB1* gene were restricted to the threonine phosphorylation site T41, with T41A being the most common variant in the pediatric (n = 8/22; 36.4%) and the adult (n = 97/170; 56.7%) DTF subgroup. Interestingly, three of the less frequent *CTNNB1* T41I variant were predominately demonstrated in pediatric DTF patients (n = 3/22; 13.6%), compared to only a single adult DTF case (n = 1/170; 0.6%) which has not been reported by other studies including pediatric DTF patients before^19,24,25^.

Crago *et al*. performed whole-exome sequencing of DTF and demonstrated additional genomic alterations in association with the canonical Wnt/β-catenin signaling pathway (i.e. loss of chromosome 6 or *BMI1* mutations), further substantiating the idea that β-catenin is the major oncogenic driver in desmoid tumor development and progression. However, in a small cohort of 26 DTF cases focusing mainly on pediatric DTF, Meazza *et al*. reported deleterious aberrations in *AKT1* (E17K; n = 8/26, 30.7%) and *BRAF* (V600E; n = 5/26,19.2%)^19^. These findings suggest a more complex mutational spectrum occurring at least in a subset of DTF than expected so far, including potentially targetable alterations that could be exploited for therapeutic benefit. To define the spectrum of non-*CTNNB1* alterations in our large DTF cohort, all protein coding exons of 26 additional cancer-associated genes were analyzed by NGS. In 15.2% of DTF cases, additional non-*CTNNB1* alterations could be detected, and 56.8% of the non-synonymous variants in *AKT1, ALK, AR, EGFR, ERBB2, IDH2, KIT, KRAS, RET, SDHA* and *SDHD* were predicted to have a potentially deleterious impact by ≥3 independent *in silico* tools. Overall, deleterious non-*CTNNB1* mutations were demonstrated in 18.2% (*AKT1, ALK, IDH2, RET* and *SDHD*) pediatric *vs*. only 8.8% (*AKT1, ALK, AR, EGFR, ERBB2, IDH2, KIT, KRAS, RET* and *SDHA*) in adult DTF cases. For further evidenced-based variant categorization, we aim to classify all detected somatic variants into four tiers based on currently available clinical and experimental evidence to determine the level of clinical significance in DTF patients for each genomic alteration as proposed in the Standards and Guidelines for the Interpretation and Reporting of Sequence Variants in Cancer^26^. In contrast to the results published by Meazza *et al*., only a single case of 22 pediatric DTF patients harbored a deleterious *AKT1* (G311D/S) mutation with unknown therapeutic relevance. A second deleterious *AKT1* (T312I) mutation was discovered in an adult DTF patient. In our entire cohort, no pathogenic *BRAF* (V600E) mutation was detected.

To the best of our knowledge, we herewith report the first DTF case with a synchronous *CTNNB1* (T41A) and imatinib-sensitive *KIT* (V559D) mutation (case #202). The tumor was localized in the Musculus rectus abdominis of a 43-year old female patient, displayed a diffusely infiltrative growth pattern and showed nuclear expression of β-catenin. The imatinib-sensitive *KIT* (V559D) alteration is known to cause ligand-independent tyrosine kinase activity and is supposed to play a central role in the pathogenesis of GIST^27–29^. If this *KIT* (V559D) mutation represented an additional oncogenic alteration in DTF, one could hypothesize that imatinib therapy might be effective. However, the clinical impact of additional deleterious mutations in the case of *CTNNB1* mutated DTF cases remains uncertain. Tyrosine kinase inhibition has been evaluated in a phase 2 study of the German Interdisciplinary Sarcoma Group (GISG-01)^16^. The authors describe a positive correlation of *CTNNB1* mutational status and progression arrest rate after imatinib treatment, especially in DTF tumors harboring a *CTNNB1* S45F alteration which were overrepresented in that study. In accordance with previously published results^30,31^, the known *KIT* polymorphism M541L was demonstrated in 16% of all analyzed DTF cases. In a prospective series by Dufresne *et al*., no predictive effect of the M541L variant on the efficacy of imatinib therapy for patients with DTF was seen^30^.

As the current study was retrospectively designed and focused on the mutational spectrum of different DTF subgroups, one of the major limitations of this study is the sparse clinical follow-up information available to us. Complementary studies comprising larger DTF cohorts are needed to correlate the mutational spectrum of both, the pediatric as well as the adult DTF subgroup to clinical outcome taking into consideration e.g. the primary localization as prognostic factor. Additional information of potential therapeutic benefit will further be gained by expanding the NGS panel used in this study (limited to the exonic region of only 27 genes) to include additional genes with targetable alterations. Furthermore, germline *APC* mutations were not validated for patients with FAP.

In conclusion, we report deleterious *CTNNB1* mutations in n = 181/204 (89%) patients with DTF confirming previous studies. Further mutational analysis indicates that a subset of DTF harbor a more complex mutational spectrum comprising not only deleterious alterations in *CTNNB1* but also potentially targetable alterations in non-Wnt/β-catenin signal transduction effectors that could be further exploited for therapeutic approaches. As an example, we herewith report the first imatinib-sensitive *KIT* (V559D) mutation in DTF which deserves further clinical investigation.

## Materials and Methods

### Tumor specimens/clinicopathological features

In total, 204 DTF tissue specimens were selected from the archive of the GIST/Sarcoma Registry (University Münster, Germany) and the Gerhard-Domagk-Institute of Pathology (Münster University Hospital, Germany). According to the current WHO classification^1^, all DTF diagnoses were reviewed by four experienced pathologists (EW, IG, SH, WH) based on morphological criteria, clinical information, immunohistochemical and molecular analyses as described before^20^. Scientific and retrospective analysis of the cohort of mesenchymal tumors was approved by the Ethics Review Board (University of Münster, 2016-091-f-S). Written informed consent from the patients was not requested and was waived by the Ethics Review Board. The study did comply with the principles set out in the United States Department of Health and Human Services Belmont Report and the World Medical Association Declaration of Helsinki. Clinicopathological data were available for 192 (94%) DTF patients (female: n = 118, 61% and male: n = 74, 39%). The cohort included 22 pediatric (≤18 y/a; mean years of age 11.9) and 170 adult (>18 y/a; mean years of age 44.9) DTF patients. With regard to clinical data, 4 cases (2%; one pediatric) were known to be associated with FAP and 200 (98%) cases were sporadic. Median tumor size was 7.0 cm (range 1-28 cm). Tumor sites were categorized into (I) extra-intestinal (n = 106, 58%), (II) intra-abdominal/mesenteric (n = 59, 32%) and (III) abdominal/abdominal wall (n = 19, 10%). All tumors within the pediatric subgroup were exclusively localized extra-intestinally. Detailed clinicopathological patient and tumor characteristics are summarized in Table 1 and individualized in Supplementary Table S1.

### Next-generation sequencing (NGS)/Sanger sequencing

A customized Qiagen GeneRead DNAseq Mix-n-Match V2 panel (in total 1028 amplicons covering 63.8 kb) was used to sequence the complete exonic region of 27 genes (*AKT1, ALK, AR, BRAF, CTNNB1, DDR2, EGFR, ERBB2, FGFR3, GNA11, GNAQ, GNAS, IDH1, IDH2, KIT, KRAS, MET, NRAS, PDGFRA, PIK3CA, PTEN, RET, SDHA, SDHB, SDHC, SDHD* and *TP53*) that are commonly mutated in cancer. As described before^32–34^, Target enrichment was conducted by means of the GeneRead DNAseq Panel PCR V2 Kit (Qiagen). Purification and size selection steps were processed utilizing Agencourt AMPure XP magnetic beads (Beckman Coulter). The GeneRead DNA Library I Core Kit (Qiagen) was applied to perform end repair, A-addition and ligation to NEXTflex-96 DNA barcodes (Bioo Scientific). The HiFi PCR Master Mix (GeneRead DNA I Amp Kit, Qiagen) and NEXTflex oligonucleotides (Bioo Scientific) were used for the amplification of adapter-ligated DNA. Sequencing was performed on the MiSeq system (Illumina; 12.5 pM library pools, 2% PhiX V3 control and MiSeq Reagent v2 chemistry). We used the Quantitative Multiplex FFPE Reference Standard (Horizon Discovery, ref.no. HD200, 11 somatic variants verified at 0.8–24.5% allelic frequency) as isogenic quality control for routine performance evaluation and monitoring of NGS workflow integrity (pre analytical DNA extraction, NGS workflow and post-analytical bioinformatics). The CLC Biomedical Genomics Workbench software (CLC bio, Qiagen) was used for NGS data analysis. Validation by Sanger sequencing (*CTNNB1* exon 3) was performed using the BigDye Terminator Cycle Sequencing Kit (v3.1, Life Technologies). Following primer set was applied: 5′-CTG ATT TGA TGG AGT TGG ACA TGG CCA TG-3′ (forward) and 5′-CCA GCT ACT TGT TCT TGA GTG AAG GAC TGA G-3′ (reverse).

### *In silico* tools to predict the potential deleterious impact of detected variants

The potential impact of NGS-detected variants in the coding regions was predicted using following *in silico* tools as reported^32–34^: PolyPhen-2 (v2.2.2r398)^35^, Protein Variation Effect Analyzer (PROVEAN; v1.1.3)^36^, Sorting Intolerant From Tolerant (SIFT; Ensembl 66)^37,38^, Mutation Assessor (release 3)^39^ and Combined Annotation Dependent Depletion (CADD; v.1.3)^40^. The PolyPhen-2 method utilizes physical and evolutionary comparative considerations to predict amino acid changes on protein structure and function. Scoring is in the range from 0 (neutral) to 1 (deleterious) and potential functional significance is categorized into benign, possibly damaging, and probably damaging. The PROVEAN web-based algorithm classified gene variants as either potentially neutral or deleterious (cutoff −2.5). SIFT was used with the default settings, classifying gene variants from 0 (damaging) to 1 (tolerated). The CADD algorithm is trained to differentiate 14.7 million high-frequency human-derived alleles for integration of diverse annotations into a single measure (C score). Scoring correlates with allelic diversity, annotations of pathogenicity, disease severity, complex trait associations, and experimentally measured regulatory effects. Calculated C scores rank a pathogenic alteration relative to all potential substitutions of the human genome. To increase the prediction accuracy and the level of confidence, a combination of *in silico* algorithms based on functional parameters, protein structure and evolutionary information was used. We combined the individual output from ≥3 of five *in silico* prediction tools and produced a single consensus outcome summarized in Supplementary Table S3.

### Immunohistochemistry (IHC)

IHC was performed on 3 µm sections from paraffin-embedded tissue using the automated Ventana BenchMark ULTRA IHC staining system (Roche). Tissue sections were deparaffinized and pre-treated with Cell Conditioning 1 solution (CC1, Ventana/Roche) for 24–64 minutes at 95–100 °C. The primary antibodies were then incubated for 16–32 minutes at 36 °C. Immunoreactions were visualized via the Optiview DAB IHC detection kit (ref.no. 760–700, Ventana/Roche). Tissue sections were counterstained with hematoxylin (ref.no. 790-2208, Ventana/Roche) and bluing solution (ref.no. 760-2037, Ventana/Roche). The mouse polyclonal β-catenin antibody (ref.no. CMC 22421040, clone 14, CellMarque) was applied. Only nuclear localization of β-catenin was considered positive for the purpose of the study, and the percentage of stained tumor cells was estimated in 10 high-power microscopic fields (400x magnification). DTF cases with ≥10% immunoreactive cells were regarded as positive. Following antibodies were additionally applied to exclude differential diagnoses: CD34 (ref.no. 790-2927, clone QBEnd10, Ventana/Roche), DOG1 (ref.no. CMC 24431050, clone SP31, CellMarque), CDK4 (ref.no. AHZ0202, DCS-31, Invitrogen/Thermo Fisher Scientific) and MDM2 (ref.no.33-7100, IF2, Invitrogen/Thermo Fisher Scientific).

### Statistical analysis

Clinical parameters of the cohort are based on descriptive statistics (comprising numbers and percentages, median and mean standard deviation). Chi-square-, Fisher’s exact- and t-tests (two-tailed with a 95% confidence interval) were calculated (SPSS 20 software, IBM and GraphPad Prism).

## Supplementary information

Supplementary Tables
