# Supplementary material for: Novel pathogenic alterations in pediatric and adult desmoid-type fibromatosis – A systematic analysis of 204 cases
Source: Sci Rep. 2020 Feb 25;10:3368. doi: 10.1038/s41598-020-60237-6 (PMC7042250; doi:10.1038/s41598-020-60237-6)
Supplement: Supplementary file 1 — Supplementary Tables [file 41598_2020_60237_MOESM1_ESM.pdf]

Novel pathogenic alterations in pediatric and adult desmoid-type fibromatosis – A systematic analysis of 204 cases

Marcel Trautmann, Jan Rehkämper, Heidrun Gevensleben, Jessica Becker, Eva Wardelmann, Wolfgang Hartmann, Inga Grünewald, and Sebastian Huss

Correspondence
Sebastian Huss MD, Gerhard-Domagk-Institute of Pathology, Münster University Hospital, Albert-Schweitzer-Campus 1, 48149 Münster, Germany
Tel: (+49) 251-83-56920, Fax: (+49) 251-83-55481
E-Mail: sebastian.huss@ukmuenster.de

Running Title
Mutational analysis of desmoid-type fibromatosis

Appendix - Table of Contents

- **Supplementary Table S1:** Clinicopathological characteristics of patients with desmoid-type fibromatosis (n=204) including genotype status

- **Supplemental Table S2:** Mutated genes in patients with desmoid-type fibromatosis

- **Supplementary Table S3:** In silico tools to predict the deleterious impact of gene variants detected in patients with desmoid-type fibromatosis (non-synonymous, ≥ 10% allelic frequency)

Supplementary Table S1: Clinicopathological characteristics of patients with desmoid-type fibromatosis (n=204) including genotype status

**Pat #**	**Age** (years) **/ gender**		**Tumor size** (cm)	**Tumor site**		***CTNNB1* mutational status**				**IHC** (nuclear β-catenin)	**Additional mutations**				
						**Coding DNA sequence change**	**Amino acid change**	**Exon**	**Allelic freq. (%)**		**Gene**	**Coding DNA sequence change**	**Amino acid change**	**Exon**	**Allelic freq. (%)**
1	41/F		ND	EI	B	121A>G	T41A	3	26	ND					
2	28/M		7	EI	D	134C>T	S45F	3	11	ND					
3	52/F		ND	EI	D	wild type				ND					
4	15/F	Infantile	ND	ND		121A>G	T41A	3	32	ND	*MET*	13G>A	A5T	2	33
5	19/M		ND	EI	B	wild type				ND					
6	43/F		9	ND		121A>G	T41A	3	32	ND					
7	30/M		6	EI	C	121A>G	T41A	3	30	ND					
8	20/F	FAP	ND	IA		wild type				ND					
9	68/M		ND	AD		121A>G	T41A	3	41	ND					
10	19/M		7.5	IA		121A>G	T41A	3	38	ND					
11	34/F		5.3	AD		121A>G	T41A	3	25	ND	*ERBB2*<br>*SDHA*	520C>T<br>112G>A	H174Y<br>D38N	4<br>2	12<br>12
12	41/F		ND	ND		wild type				ND					
13	62/M		18	EI	A	121A>G	T41A	3	25	ND					
14	18/F	Infantile	ND	EI	D	wild type				ND					
15	29/M		ND	EI	C	133T>C	S45P	3	15	ND					
16	34/M		ND	EI	C	121A>G	T41A	3	33	ND					
17	42/F		ND	EI	B	134C>T	S45F	3	34	ND					
18	42/M		ND	EI	B	133T>C	S45P	3	27	ND					
19	24/M		7.5	EI	B	121A>G	T41A	3	17	ND	*PDGFRA*	1388C>G	T463S	10	41
20	36/F		9	EI	B	134C>T	S45F	3	42	ND					
21	15/M	Infantile	ND	EI	D	133T>C	S45P	3	19	+					
22	42/F		4	IA		121A>G	T41A	3	31	+					
23	31/M		8	EI	B	134C>T	S45F	3	22	+					
24	ND		ND	ND		121A>G	T41A	3	25	+					
25	16/F	Infantile	7	EI	C	121A>G	T41A	3	45	+	*SDHD*	343C>T	R115W	4	58
26	26/F		8	EI	B	133T>A; 134C>A	S45T; S45Y	3	31	+					
27	ND		ND	ND		121A>G	T41A	3	13	+	*RET*	1025C>A	A342D	5	19
28	48/F		13	IA		121A>G	T41A	3	35	+	*ERBB2*<br>*SDHA*	520C>T<br>112G>A	H174Y<br>D38N	4<br>2	20<br>14
29	0/F	Infantile	5.3	EI	D	wild type				+	*AKT1*<br>*IDH2*	932G>A<br>931G>A<br>1060C>T	G311D<br>G311S<br>H354Y	10<br>10<br>8	29<br>16<br>25
30	7/M	Infantile	3.5	EI	B	122C>T	T41I	3	17	+					
31	ND		ND	ND		134C>T	S45F	3	16	ND	*EGFR*	3187G>A<br>3191G>A	D1063N<br>S1064N	27<br>27	12<br>10
32	38/F		ND	EI	C	121A>G	T41A	3	7	ND	*AKT1*	935C>T	T312I	10	27
33	4/M	Infantile, FAP	3.5	EI	A	wild type				ND					
34	ND		ND	ND		122C>T	T41I	3	34	ND	*ERBB2*<br>*IDH2*	520C>T<br>1060C>T	H174Y<br>H354Y	4<br>8	18<br>16
35	2/M	Infantile	3	EI	A	wild type				ND	*ALK*	2770G>A	G924S	16	19
36	33/F		5	AD		133T>C	S45P	3	22	+					
37	25/M		13	EI	B	133T>C; 134C>T	S45P; S45F	3	27	+	*AR*<br>*SDHA*	475G>A<br>107C>T	A159T<br>T36I	1<br>2	74<br>17
38	ND		ND	ND		133T>C; 134C>T	S45P; S45F	3	25	ND	*AR*	475G>A	A159T	1	75
39	68/M		9.5	IA		121A>G	T41A	3	41	+					
40	43/F		5.2	IA		133T>C	S45P	3	17	+					
41	35/F		6	EI	A	121A>G	T41A	3	17	ND					
42	47/F		27	AD		wild type				ND					
43	27/F		ND	IA		121A>G	T41A	3	32	ND	*EGFR*<br>*KRAS*	3173G>A<br>531_533delGAA	C1058Y<br>K180del	27<br>5	10<br>52
44	1/F	Infantile	ND	EI	A	122C>T	T41I	3	34	ND					
45	ND		ND	ND		134C>T	S45F	3	20	ND					
46	53/F		ND	EI	B	122C>T	T41I	3	13	ND					
47	42/M		ND	EI	D	wild type				ND					
48	ND		ND	ND		134C>T	S45F	3	44	ND					

49	14/F	Infantile, Autism	8	EI	C	134C>T	S45F	3	44	ND					
50	51/F		10.5	IA		121A>G	T41A	3	30	ND					
51	74/M		10.5	AD		133delinsAAGG	S45KA	3	37	ND					
52	ND		ND	ND		121A>G	T41A	3	35	ND					
53	61/M		3.8	IA		wild type				ND					
54	35/F		ND	EI	D	133T>C	S45P	3	23	ND					
55	ND		ND	ND		133T>C	S45P	3	21	ND					
56	63/F		2.2	EI	B	121A>G	T41A	3	28	ND					
57	28/F		10	EI	D	121A>G	T41A	3	58	ND					
58	50/F		ND	EI	A	121A>G	T41A	3	31	ND					
59	ND		ND	ND		121A>G	T41A	3	41	ND					
60	23/M		ND	EI	D	121A>G	T41A	3	18	ND					
61	ND		ND	ND		121A>G	T41A	3	27	ND					
62	ND		ND	ND		121A>G	T41A	3	41	ND					
63	60/F		ND	EI	ND	121A>G	T41A	3	49	ND					
64	73/F		ND	EI	D	133T>C	S45P	3	8	ND					
65	30/M		8	EI	C	121A>G	T41A	3	22	ND					
66	16/M	Infantile	ND	EI	D	121A>G	T41A	3	36	ND					
67	35/M		ND	EI	B	121A>G	T41A	3	16	ND					
68	15/M	Infantile	7	EI	D	121A>G	T41A	3	18	ND					
69	16/F	Infantile	ND	EI	D	121A>G	T41A	3	19	ND					
70	37/F		5	AD		133T>C	S45P	3	28	ND					
71	16/F	Infantile	4.5	EI	C	121A>G	T41A	3	26	ND					
72	53/F		ND	EI	D	134C>T	S45F	3	41	ND					
73	26/F		ND	EI	B	121A>G	T41A	3	37	ND					
74	19/M		11.7	IA		121A>G	T41A	3	23	+	*PDGFRA*	1480G>C	E494Q	10	13
75	53/F		7	IA		121A>G	T41A	3	39	+					
76	58/F		2.5	IA		121A>G	T41A	3	24	+					
77	74/F		ND	ND		134C>T	S45F	3	41	+					
78	73/M		12	IA		121A>G	T41A	3	33	+					
79	36/F		11.5	IA		121A>G	T41A	3	37	+	*SDHA*	974G>T	R325M	8	51
80	43/F		6	AD		121A>G	T41A	3	31	ND					
81	28/M		ND	EI	B	134C>T	S45F	3	31	+					
82	70/M		ND	EI	B	wild type				+					
83	72/F		3	IA		133T>C	S45P	3	6	+					
84	50/F		ND	IA		121A>G	T41A	3	47	+					
85	33/F		ND	AD		wild type				+					
86	27/F		ND	EI	B	121A>G	T41A	3	33	+	*EGFR*	1580G>A	R527Q	13	53
87	60/F		5	IA		121A>G	T41A	3	42	+					
88	73/F		ND	EI	A	121A>G	T41A	3	43	ND					
89	25/F		ND	EI	D	121A>G	T41A	3	18	+					
90	9/M	Infantile	ND	EI	A	134C>T	S45F	3	29	+	*RET*	3112A>G	T1038A	19	55
91	28/F		ND	EI	D	134C>T	S45F	3	43	+	*KRAS*	565A>C	M189L	5	48
92	34/M		ND	EI	D	134C>T	S45F	3	18	+					
93	47/M		6.5	IA		134C>T	S45F	3	17	+					
94	45/F		3.4	EI	C	121A>G	T41A	3	28	+					
95	27/F		ND	EI	D	121A>G	T41A	3	25	+					
96	45/F		ND	EI	B	wild type				+					
97	69/M		8	EI	B	121A>G	T41A	3	32	+					
98	62/F		5.5	IA		121A>G	T41A	3	24	+					
99	44/F		ND	EI	C	121A>G	T41A	3	31	+	*RET*	1538C>T	A513V	8	52
100	56/F		3.5	EI	B	121A>G	T41A	3	30	+	*AR*	1889G>A	R630Q	4	15
101	64/F		9.5	IA		134C>T	S45F	3	32	+					
102	32/F		9	AD		121A>G	T41A	3	38	ND					
103	22/M		ND	EI	D	134C>T	S45F	3	30	+					
104	38/F		ND	AD		121A>G	T41A	3	37	+	*KIT*	646G>C	V216L	4	16
105	20/F		28	IA		133T>C	S45P	3	34	+					
106	40/F		ND	EI	B	121A>G	T41A	3	41	+					
107	56/M		14	IA		121A>G	T41A	3	35	+					
108	14/F	Infantile	4.5	EI	D	134C>T	S45F	3	32	+					
109	45/F		ND	IA		121A>G	T41A	3	38	+					
110	46/F		ND	ND		121A>G	T41A	3	19	ND					
111	41/M		ND	EI	B	134C>T	S45F	3	18	+					
112	73/M		ND	EI	B	121A>G	T41A	3	39	+					
113	30/F		ND	EI	C	134C>T	S45F	3	21	+					

114	42/M		9	IA		121A>G	T41A	3	43	+					
115	40/F		1	IA		121A>G	T41A	3	51	+	*ALK*	3140C>G	A1047G	19	47
116	53/F		ND	EI	B	121A>G	T41A	3	24	+					
117	53/M		ND	IA		121A>G	T41A	3	28	+					
118	62/M		ND	IA		134C>T	S45F	3	21	+					
119	43/F		4.8	AD		97T>A; 100G>C	S33T; G34R	3	25	+					
120	63/M		4	IA		121A>G	T41A	3	14	ND					
121	31/F		ND	EI	D	133T>C	S45P	3	30	+					
122	33/F		22.5	EI	B	121A>G	T41A	3	41	+	*ALK*	780C>A	S260R	2	49
123	34/F		7.7	AD		134C>T	S45F	3	36	+					
124	69/M		ND	EI	A	134C>T	S45F	3	32	+					
125	27/F		ND	EI	D	134C>T	S45F	3	28	+					
126	20/F		ND	EI	B	134C>T	S45F	3	20	+					
127	52/M		9	IA		133T>C	S45P	3	16	+					
128	71/M		ND	IA		121A>G	T41A	3	30	+					
129	50/F		18	IA		121A>G	T41A	3	36	+					
130	26/F		2.7	EI	D	121A>G	T41A	3	32	+					
131	82/F		4.8	EI	A	wild type				+					
132	33/F		ND	AD		121A>G	T41A	3	26	+					
133	55/M		ND	IA		121A>G	T41A	3	28	+					
134	40/M		ND	IA		wild type				+					
135	72/F		5	EI	A	121A>G	T41A	3	7	+					
136	73/M		7.5	IA		134C>T	S45F	3	19	+					
137	29/M		8.5	IA		121A>G	T41A	3	24	+					
138	1/M	Infantile	ND	EI	A	122C>T	T41I	3	30	+					
139	37/F	FAP	13	IA		wild type				ND					
140	20/F		ND	EI	B	121A>G	T41A	3	37	+					
141	14/F	Infantile	ND	EI	B	wild type				+					
142	58/M		ND	IA		121A>G	T41A	3	27	ND					
143	29/F		ND	EI	B	121A>G	T41A	3	29	+					
144	18/F	Infantile	9.5	EI	D	134C>T	S45F	3	29	+					
145	40/F		ND	EI	A	134C>T	S45F	3	19	+					
146	28/F		ND	EI	D	134C>T	S45F	3	42	ND					
147	52/F		ND	EI	B	134C>T	S45F	3	21	+	*ALK*	2417G>A	R806H	14	34
148	80/M		14.5	IA		121A>G	T41A	3	29	+	*RET*	3112A>G	T1038A	19	47
149	32/F		ND	AD		133T>A; 134C>T	S45T; S45F	3	15	+					
150	55/F		1.1	EI	ND	wild type				ND					
151	29/M		7	IA		121A>G	T41A	3	22	ND					
152	25/F		9	EI	B	134C>T	S45F	3	20	+					
153	17/F	Infantile	ND	EI	B	121A>G	T41A	3	31	+					
154	80/F		ND	EI	B	121A>G	T41A	3	17	+					
155	65/F		12	IA		121A>G	T41A	3	32	+					
156	68/M		1	EI	ND	133T>C	S45P	3	13	+					
157	61/M		ND	IA		121A>G	T41A	3	29	+					
158	47/F		ND	EI	B	134C>T	S45F	3	29	ND					
159	21/F		ND	EI	D	121A>G	T41A	3	42	+					
160	32/F		ND	EI	B	121A>G	T41A	3	24	+					
161	25/F		10.7	IA		121A>G	T41A	3	38	+					
162	42/M		15	IA		133T>C	S45P	3	41	+					
163	16/F	Infantile	ND	ND		134C>T	S45F	3	25	ND					
164	47/F		ND	EI	B	121A>G	T41A	3	10	+					
165	45/M		ND	EI	B	133T>C	S45P	3	23	ND					
166	60/M		11.5	IA		121A>G	T41A	3	46	+					
167	37/F		ND	AD		121A>G	T41A	3	28	+					
168	32/F		1.7	AD		133T>C	S45P	3	20	+					
169	50/F		ND	EI	B	121A>G	T41A	3	32	+	*FGFR3*	2302C>T	P768S	17	25
170	55/M		ND	IA		121A>G	T41A	3	17	+					
171	23/F		ND	EI	B	121A>G	T41A	3	19	+					
172	39/F		ND	ND		133T>C	S45P	3	39	+					
173	41/M		3.1	EI	B	wild type				+					
174	43/F		3.5	EI	B	121A>G	T41A	3	26	+					
175	70/M		ND	IA		121A>G	T41A	3	32	+					
176	51/M		ND	EI	B	134C>T	S45F	3	33	+					
177	21/F		ND	EI	D	134C>T	S45F	3	28	+					
178	34/F		ND	EI	D	121A>G	T41A	3	40	+					
179	61/M		3	IA		121A>G	T41A	3	40	+	*MET*	3352A>G	I1118V	17	53

180	55/M		9	IA		121A>G	T41A	3	32	+					
181	60/M		7.5	IA		97_98delinsCT	S33L	3	26	+					
182	48/M		ND	EI	B	134C>T	S45F	3	31	+					
183	47/F		ND	EI	B	wild type				+					
184	28/F		ND	EI	B	134C>T	S45F	3	33	+					
185	17/F	Infantile	12.5	EI	D	121A>G	T41A	3	21	+					
186	67/M		14	IA		121A>G	T41A	3	30	+					
187	43/M		4.5	IA		121A>G	T41A	3	7	+					
188	31/F		ND	EI	B	wild type				+					
189	29/M		2	IA		121A>G	T41A	3	20	+					
190	26/F	FAP	ND	IA		wild type				+					
191	28/F		12.5	EI	D	121A>G	T41A	3	23	+					
192	40/F		ND	AD		121A>G	T41A	3	36	+					
193	56/F		6.6	EI	D	121A>G	T41A	3	24	+	*IDH2*	1060C>T	H354Y	8	12
194	52/M		11.2	IA		121A>G	T41A	3	11	+					
195	52/M		6	IA		121A>G	T41A	3	17	+	*EGFR*	2543C>T	P848L	21	48
196	75/M		10	IA		121A>G	T41A	3	43	+					
197	45/F		ND	EI	D	134C>T	S45F	3	24	+					
198	78/F		5	EI	B	121A>G	T41A	3	21	+					
199	77/M		ND	EI	B	134C>T	S45F	3	26	+					
200	23/M		ND	IA		134C>T	S45F	3	28	+					
201	35/F		4.2	AD		121A>G	T41A	3	19	+					
202	43/F		2.5	AD		121A>G	T41A	3	25	+	*KIT*	1676T>A	V559D	11	18
203	53/F		4.8	IA		121A>G	T41A	3	36	+					
204	73/M		5.7	IA		121A>G	T41A	3	37	+					

Abbreviations: *F*, female; *M*, male; *FAP*, familial adenomatous polyposis coli; *ND*, not determined; *EI*, extra-intestinal; *IA*, intra-abdominal; *AD*, abdominal; *A*; head/neck; *B*, trunk; *C*, upper extremities; *D*, lower extremities; *IHC*, immunohistochemistry

Supplemental Table S2: Mutated genes in patients with desmoid-type fibromatosis

Gene	[A] Approved gene name	[A] HGNC ID	[B] Entrez Gene ID	[C] OMIM ID	Chr. location	Chr. strand	[D] Transcript ID (Ensembl release 90, Human GRCh38.p10)	[E] RefSeq	[D] Protein ID (Ensembl release 90, Human GRCh38.p10)	[F] UniProtKB
AKT1	AKT Serine/Threonine Kinase 1	391	207	164730	14q32.33	reverse	ENST00000555528	NM_005163	ENSP00000450688	P31749
ALK	Anaplastic Lymphoma Receptor Tyrosine Kinase	427	238	105590	2p23.2-p23.1	reverse	ENST00000389048	NM_004304	ENSP00000373700	Q9UM73
AR	Androgen receptor	644	367	313700	Xq12	forward	ENST00000374690	NM_000044	ENSP00000363822.3	P10275
CTNNB1	Catenin Beta 1	2514	1499	116806	3p22.1	forward	ENST00000396183	NM_001098210	ENSP00000379486	P35222
EGFR	Epidermal Growth Factor Receptor	3236	1956	131550	7p11.2	forward	ENST00000275493<br>ENST00000442591	NM_005228	ENSP00000275493<br>ENSP00000410031	P00533
ERBB2	Erb-B2 Receptor Tyrosine Kinase 2	3430	2064	164870	17q12	forward	ENST00000269571	NM_004448	ENSP00000269571	P04626
FGFR3	Fibroblast Growth Factor Receptor 3	3690	2261	134934	4p16.3	forward	ENST00000481110	NM_001354810	ENSP00000420533	F8W9L4
IDH2	Isocitrate dehydrogenase (NADP(+)) 2	5383	3418	147650	15q26.1	reverse	ENST00000330062	NM_002168	ENSP00000331897	P48735
KIT	KIT Proto-Oncogene Receptor Tyrosine Kinase	6342	3815	164920	4q12	forward	ENST00000288135	NM_000222	ENSP00000288135	P10721
KRAS	KRAS proto-oncogene	6407	3845	190070	12p12.1	reverse	ENST00000256078<br>ENST00000311936	NM_033360<br>NM_004985	ENSP00000256078<br>ENSP00000308495	P01116
MET	MET proto-oncogene	7029	4233	164860	7q31	forward	ENST00000397752	NM_000245	ENSP00000380860	P08581
PDGFRA	Platelet derived growth factor receptor alpha	8803	5156	173490	4q12	forward	ENST00000257290	NM_006206	ENSP00000257290	P16234
RET	Ret proto-oncogene	9967	5979	164761	10q11.21	forward	ENST00000355710	NM_020975	ENSP00000347942	P07949
SDHA	Succinate dehydrogenase complex flavoprotein subunit A	10680	6389	600857	5p15.33	forward	ENST00000264932	NM_004168	ENSP00000264932	P31040
SDHD	Succinate dehydrogenase complex flavoprotein subunit D	10683	6390	602690	11q23.1	forward	ENST00000526592	NM_001276506	ENSP00000432005	O14521

[A] **HGNC** - HUGO Gene Nomenclature Committee (http://www.genenames.org/)
[B] **Entrez Gene** (https://www.ncbi.nlm.nih.gov/gene)
[C] **OMIM** - Online Mendelian Inheritance in Man (http://omim.org/)
[D] **Ensembl** (http://www.ensembl.org/index.html)
[E] **RefSeq** - Reference Sequence Database hosted by the National Center for Biotechnology Information (NCBI) (https://www.ncbi.nlm.nih.gov/refseq/)
[F] **UniProt** - The Universal Protein Resource (http://www.uniprot.org/)

Supplementary Table S3: *In silico* tools to predict the deleterious impact of gene variants detected in patients with desmoid-type fibromatosis (non-synonymous, ≥ 10% allelic frequency)

Pat. #	Gene	Chromosom	Exon	Region (GRCh38/ hg38)	Coding DNA sequence change	Amino acid change	[A] COSMIC ID (COSM; v82)	[B] PolyPhen-2 (v2.2.2r398)		[C] PROVEAN prediction (v1.1.3)		[D] SIFT (Ensembl 66)		[E] Mutation Assessor (release 3)		[F] Combined Annotation Dependent Depletion (CADD, v.1.3)	
								pph2 prob	prediction	score	prediction (cutoff = - 2.5)	score	prediction (cutoff = 0.05)	FI score	functional impact	PHRED like C-score	category
29	***AKT1***	14	10	104773277	931G>A	**G311S**		0.95	possibly damaging	-5.80	Deleterious	0.000	Damaging	3.25	medium	27.7	1%
29	***AKT1***	14	10	104773276	932G>A	**G311D**	COSM2026428	0.998	probably damaging	-6.76	Deleterious	0.000	Damaging	4.165	high	25.8	1%
32	***AKT1***	14	10	104773273	935C>T	**T312I**		0.997	probably damaging	-5.53	Deleterious	0.000	Damaging	3.595	high	24.4	1%
122	***ALK***	2	2	29717585	780C>A	**S260R**		0.917	possibly damaging	-0.72	Neutral	0.062	Tolerated	0.55	neutral	24.2	1%
147	***ALK***	2	14	29233635	2417G>A	**R806H**		0.991	probably damaging	-1.94	Neutral	0.107	Tolerated	2.47	medium	31	0.1%
35	***ALK***	2	16	29228929	2770G>A	**G924S**		1	probably damaging	-4.05	Deleterious	0.099	Tolerated	3.745	high	33	0.1%
115	***ALK***	2	19	29225493	3140C>G	**A1047G**		0.002	benign	-0.34	Neutral	0.330	Tolerated	0.945	low	22.1	1%
37, 38	***AR***	X	1	67545621	475G>A	**A159T**		ND	ND	-1.00	Neutral	0.002	Damaging	ND	ND	27.8	1%
100	***AR***	X	4	67711405	1889G>A	**R630Q**	COSM4962608	ND	ND	-3.14	Deleterious	0.001	Damaging	ND	ND	32	0.1%
181	***CTNNB1***	3	3	41224609.. 41224610	97_98delinsCT	**S33L**	COSM6098	1	probably damaging	-4.82	Deleterious	0.006	Damaging	2.66	medium	ND	ND
119	***CTNNB1***	3	3	41224609	97T>A	**S33T**	COSM27311	1	probably damaging	-2.30	Neutral	0.002	Damaging	2.66	medium	25.0	1%
119	***CTNNB1***	3	3	41224612	100G>C	**G34R**	COSM5684	1	probably damaging	-6.20	Deleterious	0.000	Damaging	2.66	medium	26.8	1%
1+	***CTNNB1***	3	3	41224633	121A>G	**T41A**	COSM5664	0.94	possibly damaging	-3.36	Deleterious	0.003	Damaging	2.68	medium	26.1	1%
30+	***CTNNB1***	3	3	41224634	122C>T	**T41I**	COSM5676	0.996	probably damaging	-4.19	Deleterious	0.001	Damaging	2.68	medium	27.3	1%
51	***CTNNB1***	3	3	41224645	133delinsAAGG	**S45KA**		ND	ND	-7.28	Deleterious	ND	ND	2.565	medium	15.30	10%
149	***CTNNB1***	3	3	41224645	133T>A	**S45T**	COSM5719	0.132	benign	-1.92	Neutral	0.033	Damaging	2.215	medium	18.22	10%
15+	***CTNNB1***	3	3	41224645	133T>C	**S45P**	COSM5663	0.988	probably damaging	-2.93	Deleterious	0.002	Damaging	2.215	medium	25.7	1%
26	***CTNNB1***	3	3	41224646	134C>A	**S45Y**	COSM5692	0.999	probably damaging	-3.62	Deleterious	0.000	Damaging	2.565	medium	27.6	1%
2+	***CTNNB1***	3	3	41224646	134C>T	**S45F**	COSM5667	0.996	probably damaging	-3.85	Deleterious	0.000	Damaging	2.565	medium	28.3	1%
86	***EGFR***	7	13	55161580	1580G>A	**R527Q**	COSM4680394	0.007	benign	-1.48	Neutral	0.168	Tolerated	0.945	low	0.137	/
195	***EGFR***	7	21	55191792	2543C>T	**P848L**	COSM22943	1	probably damaging	-8.85	Deleterious	0.001	Damaging	0.37	neutral	33	0.1%
43	***EGFR***	7	27	55202527	3173G>A	**C1058Y**		0	benign	-1.07	Neutral	1.000	Tolerated	0.805	low	1.112	/
31	***EGFR***	7	27	55202541	3187G>A	**D1063N**		0.041	benign	-1.33	Neutral	0.236	Tolerated	1.62	low	16.71	10%
31	***EGFR***	7	27	55202545	3191G>A	**S1064N**		0	benign	-1.66	Neutral	0.018	Damaging	2.195	medium	22.9	1%
11, 28, 34	***ERBB2***	17	4	39709398	520C>T	**H174Y**		0.778	possibly damaging	-1.55	Neutral	0.019	Damaging	2.08	medium	23.3	1%
169	***FGFR3***	4	17	1807211	2302C>T	**P768S**		0.001	benign	-0.20	Neutral	0.000	Damaging	ND	ND	15.94	10%
29, 34, 193	***IDH2***	15	8	90085295	1060C>T	**H354Y**		0.995	probably damaging	-5.31	Deleterious	0.000	Damaging	4.14	high	25.4	1%
104	***KIT***	4	4	54699656	646G>C	**V216L**		0	benign	1.27	Neutral	1.000	Tolerated	0.435	neutral	0.180	/

202	***KIT***	4	11	54727444	1676T>A	**V559D**	COSM1252	1	probably damaging	-5.77	Deleterious	0.000	Damaging	2.87	medium	33	0.1%
91	***KRAS***	12	5	25215446	565A>C	**M189L**		0	benign	-0.16	Neutral	0.867	Tolerated	-0.445	neutral	12.64	10%
4	***MET***	7	2	116699097	13G>A	**A5T**		0.017	benign	0.20	Neutral	0.527	Tolerated	1.1	low	4.5	/
179	***MET***	7	17	116778787	3352A>G	**I1118V**		0.033	benign	-0.35	Neutral	0.185	Tolerated	0.13	neutral	22.9	1%
19	***PDGFRA***	4	10	54273560	1388C>G	**T463S**		0.004	benign	-1.13	Neutral	0.138	Tolerated	0.825	low	4.8	/
74	***PDGFRA***	4	10	54273652	1480G>C	**E494Q**		0.629	possibly damaging	-0.48	Neutral	0.198	Tolerated	1.555	low	12.99	10%
27	***RET***	10	5	43106533	1025C>A	**A342D**		0.282	benign	-0.99	Neutral	0.148	Tolerated	2.08	medium	17.96	10%
99	***RET***	10	8	43112114	1538C>T	**A513V**	COSM3375464	0.007	benign	-0.05	Neutral	0.389	Tolerated	0.69	neutral	7.2	/
90, 148	***RET***	10	19	43126647	3112A>G	**T1038A**	COSM4650197	0.968	probably damaging	-3.01	Deleterious	0.004	Damaging	0.695	neutral	25.8	1%
37	***SDHA***	5	2	223525	107C>T	**T36I**		ND	ND	-1.36	Neutral	0.094	Tolerated	1.845	low	18.67	10%
11, 28,	***SDHA***	5	2	223530	112G>A	**D38N**		ND	ND	0.30	Neutral	0.690	Tolerated	0.315	neutral	9.1	/
79	***SDHA***	5	8	233555	974G>T	**R325M**		ND	ND	-5.48	Deleterious	0.000	Damaging	5.11	high	29.6	1%
25	***SDHD***	11	4	112093108	343C>T	**R115W**		ND	ND	-1.16	Neutral	0.007	Damaging	ND	ND	0.340	/

[A] ***COSMIC*** - Catalogue of somatic mutations in cancer (http://cancer.sanger.auk/cosmic)
[B] **PolPhen-2**: Prediction outcome can be *benign*, *possibly damaging*, or *probably damaging* – (http://genetics.bwh.harvard.edu/pph2/bgi.shtml)
[C] **PROVEAN**: Prediction outcome can be *deleterious* or *neutral* (cutoff = -2.5) – (http://provean.jcvi.org/protein_batch_submit.php?species=human)
[D] **SIFT**: Prediction outcome can be *tolerated* or *damaging* (cutoff = 0.05) – (http://provean.jcvi.org/protein_batch_submit.php?species=human)
[E] **Mutation Assessor**: Functional impact of a variant is described as predicted functional (*high, medium*) or predicted non-functional (*low, neutral*) – (http://mutationassessor.org/r3/)
[F] **Combined Annotation Dependent Depletion (CADD)**: PHRED-like scaled C-scores rank a variant relative to all possible substitutions of the human genome. A scaled C-score ≥10 indicates that these variants are predicted to be amongst the 10% most deleterious substitutions in the human genome, ≥20 = 1% and ≥30 = 0.1% – (http://cadd.gs.washington.edu/)
